# Supplementary figures and images for: A gene expression study of dorso-ventrally restricted pigment pattern in adult fins of Neolamprologus meeli, an African cichlid species
Source: PeerJ. 2017 Jan 10;5:e2843. doi: 10.7717/peerj.2843 (PMC5228514; doi:10.7717/peerj.2843)

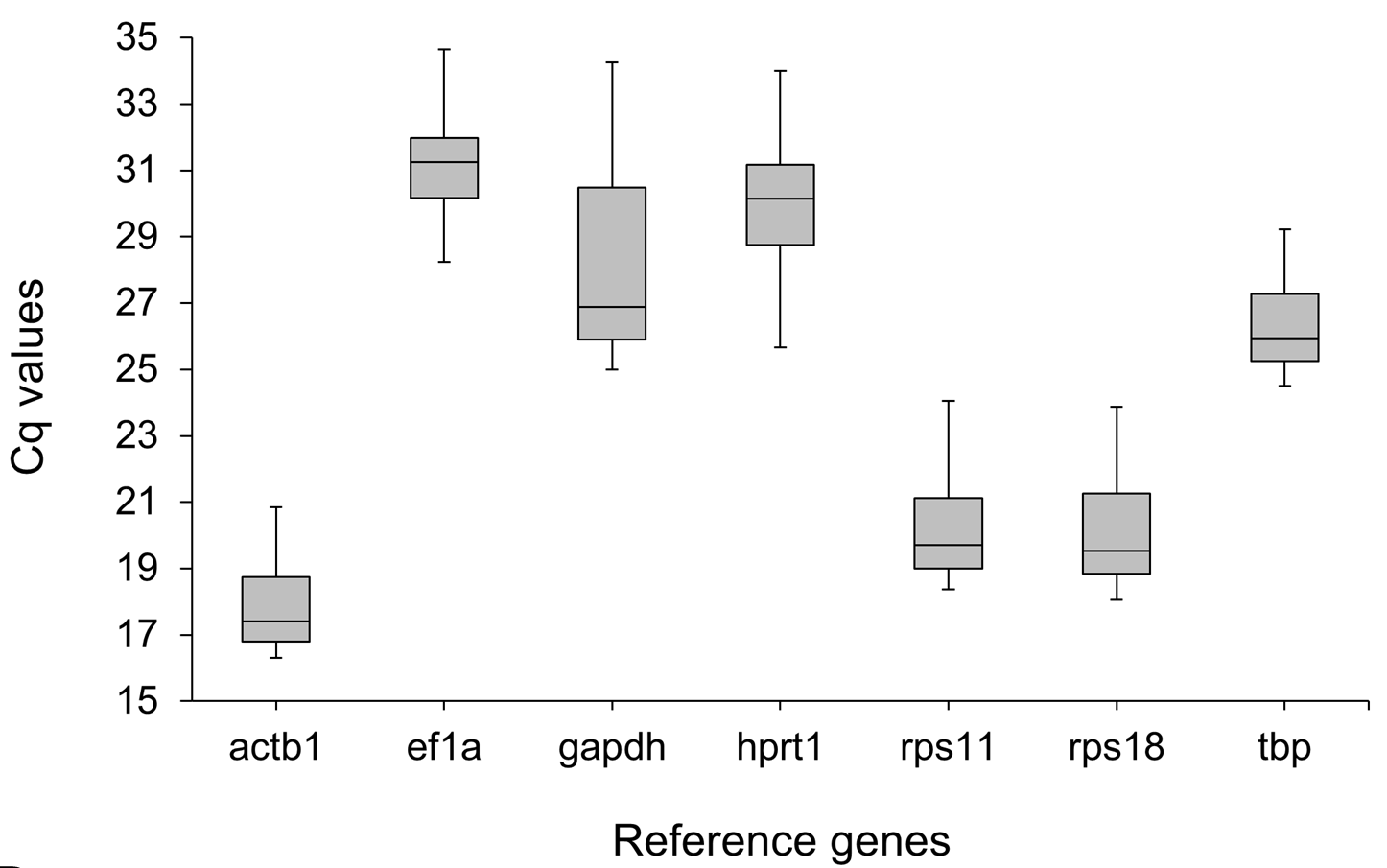

Supplement: Figure S1 — Expression profiles of candidate reference genes in raw Cq values for all samples (12 fin samples for each of 4 adult fishes). The middle line denotes the median and boxes indicate the 25/75 percentiles. [file peerj-05-2843-s003.png]

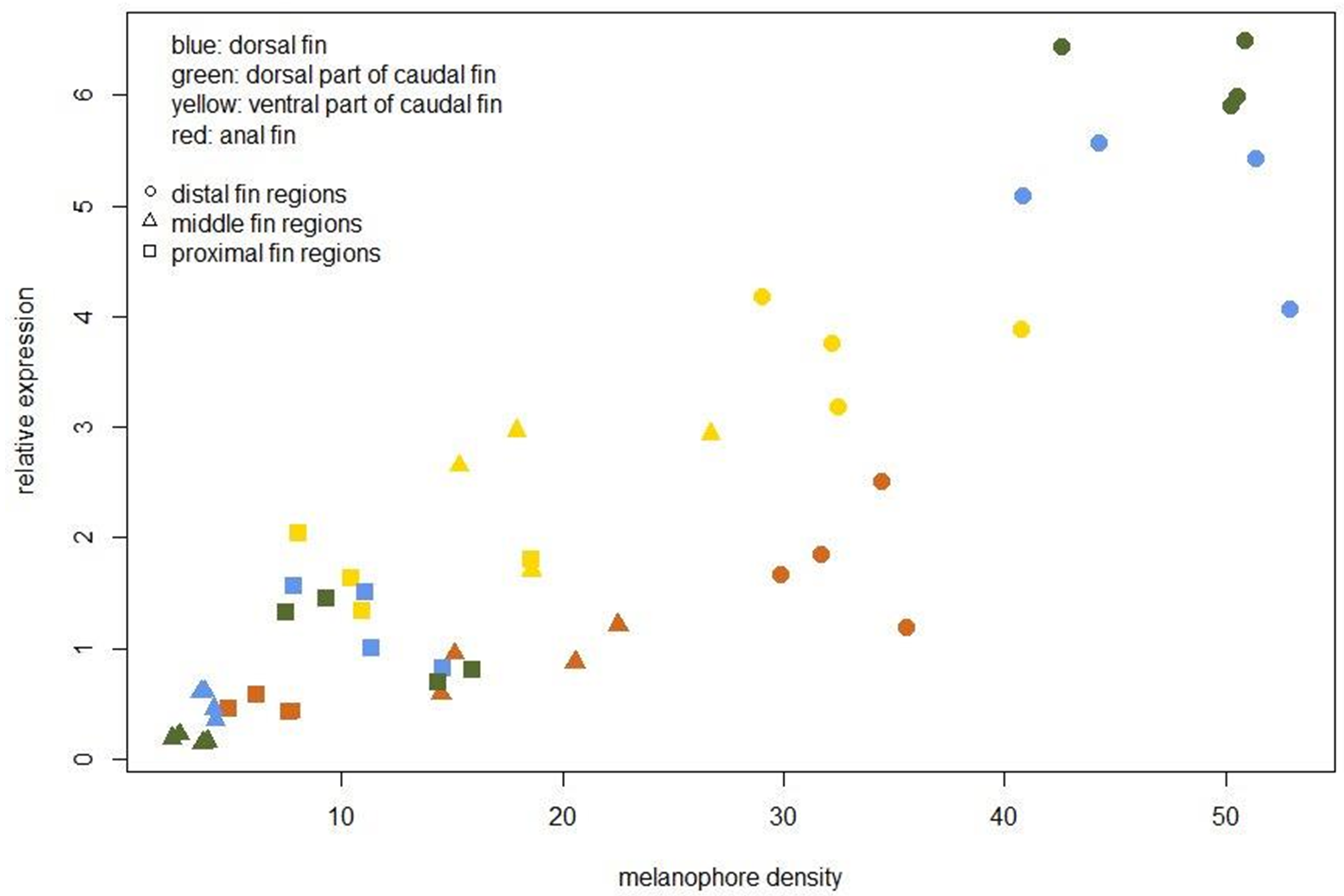

Supplement: Figure S2 — The fins and anatomical regions within fins are coded by different colours and symbols, respectively. [file peerj-05-2843-s004.png]
